# Lack of association between serum homocysteine level and middle cerebral artery stenosis

**DOI:** 10.1002/brb3.1297

**Published:** 2019-06-21

**Authors:** Haitao Zhou, Chao Huang, Ruihua Liu, Chao Liu, Congmin Ma, Xiangyang Ren

**Affiliations:** ^1^ Neurology Department Luoyang Central Hospital Affiliated to Zhengzhou University Luoyang China

**Keywords:** homocysteine, ischemic stroke, middle cerebral artery, stenosis

## Abstract

**Objectives:**

Lowering homocysteine (HCY) has beneficial effects on vascular events in primary prevention but not in secondary prevention. Research on serum HCY level and middle cerebral artery (MCA) stenosis is lacking. The purpose of the study was to determine the association between these factors and provide more evidence for the prevention of ischemic stroke.

**Methods:**

A total of 412 patients (35–93 years old) in the Neurology Department were recruited. Data of clinical and biochemical vascular risk factors were collected. MCA stenosis, including M1, M2, and M3, was determined by brain magnetic resonance angiography (MRA) and classified into stenosis or no stenosis. The differences and associations were analyzed by relevant statistical methods.

**Results:**

There was no significant difference (*p *=* *0.325) in HCY levels between the MCA stenosis and no stenosis groups at baseline. Logistic regression analysis demonstrated that there was no significant association between HCY levels and MCA stenosis (*p *=* *0.447). After the two groups were matched for age and sex, there was still no difference (*p *=* *0.540 for males and 0.061 for females) or association (*p *=* *0.709 for males and 0.098 for females). In addition, we found that ischemic stroke was more prevalent in the MCA stenosis group and uric acid was higher in males with MCA stenosis.

**Conclusions:**

The study indicates a lack of association between serum HCY level and MCA stenosis, which may partially explain the negative results of secondary prevention clinical trials focused on lowering serum HCY level. Future studies on HCY reduction should focus more on primary prevention of ischemic stroke.

## 1 INTRODUCTION

Lowering homocysteine (HCY) is a sulfur‐containing amino acid produced by demethylation of the essential amino acid methionine (Dennis, Nurko, & Robinson, 1997). Several studies have confirmed that elevated serum HCY level is a risk factor for cardiovascular and cerebrovascular diseases (Dikmen et al., 2006; Kwon, Lee, Bae, & Kang, 2014; Wald, Law, & Morris, 2002; Yokote, Shiraishi, Shintani, & Shiigai, 2007). A persistent elevated serum HCY level damages endothelial cells in the cerebral artery, gradually induces atherosclerosis to stenosis and eventually leads to ischemic stroke (Clarke et al., 1991; McCully, 1969; Zhang et al., 2012).

Middle cerebral artery (MCA) stenosis leading to cerebral infarction characterized by impaired motor and/or language function is an important form of cerebral arteriosclerosis (Jeng et al., 2017). A previous study proposed that elevated serum HCY level is associated with MCA stenosis (Huang et al., 2007). However, several secondary prevention clinical trials have revealed that reductions in serum HCY level with B vitamins and folic acid supplementation did not have beneficial effects on vascular outcomes (Armitage et al., 2010; Bonaa et al., 2006; Lonn et al., 2006). Therefore, the association between serum HCY level and MCA stenosis may be complicated.

Given the lack of research on the association between serum HCY level and MCA stenosis, the purpose of this cross‐sectional study was to further investigate the association and provide more evidence for prevention of ischemic stroke.

## 2 METHODS

### 2.1 Subjects

A total of 412 patients (35–93 years old) in the Neurology Department were included in this study from June 2017 to September 2017 in the Luoyang Central Hospital affiliated to Zhengzhou University. Inclusion criteria were as follows: integrated medical records and valid MCA imaging. Exclusion criteria were as follows: medical records deficiency, tumors, severe malnutrition and kidney failure. All subjects signed informed consent forms before the study. The study was approved by the ethics committee of the Luoyang Central Hospital.

### 2.2 Clinical vascular risk factors

All data were collected from the integrated medical records of inpatients in the Neurology Department. Smoking status was recorded as never or not in males. Hypertension was diagnosed when blood pressure was ≥140/90 mmHg or with a self‐reported history of hypertension or current antihypertensive treatment. Diabetes mellitus was confirmed in patients with fasting blood glucose levels >7.0 mmol/L or with a self‐reported history of diabetes or antidiabetic drug or insulin use. Coronary heart disease was confirmed in patients with a self‐reported history and antiplatelet drug use. Atrial fibrillation was diagnosed using electrocardiogram and stethoscope or a self‐reported history or anticoagulant drug use. Ischemic stroke was confirmed in patients with a self‐reported history and antiplatelet drug use.

### 2.3 Biochemical vascular risk factors

Serum biochemical vascular risk factors, such as total cholesterol (TC), triglyceride (TG), low‐density lipoprotein cholesterol (LDL‐C), fasting blood glucose, HbA1c, uric acid, and HCY were measured using standard laboratory techniques.

### 2.4 Brain MRA

Brain MRA was performed in all subjects using a 3.0 T machine (Magnetom Verio, Siemens AG), with six slabs, each including 40 axial slices of 0.5‐mm thickness and no interslice gaps. The 3D TOF images were acquired with the following parameters: TR/TE, 21/3.6 ms; flip angle, 15°; field of view, 219 × 219 mm; matrix number, 768 × 768; and axial slice thickness, 0.7 mm (Chen et al., 2015). MCA, including M1, M2, and M3, was classified into stenosis or no stenosis.

### 2.5 Statistical analyses

The data are described as the mean ± standard deviations (*SD*) for quantitative data, frequencies, and percent for qualitative data. The differences in factors in two groups were assessed using chi‐square tests or Student's *t*‐tests according to the data type. The association between MCA stenosis and the indicated variables was analyzed using bivariate logistic regression analysis. Data were analyzed using the Statistical Package for the Social Sciences (SPSS) 17.0 on Windows 7.0. *p *<* *0.05 was considered statistically significant.

## 3 RESULTS

### 3.1 Difference in the indicated factors between the no MCA stenosis group and the MCA stenosis group at baseline

Four hundred and twelve patients (209 males and 203 females; mean age 66.29 years old) were classified into two groups: the no MCA stenosis group (*n* = 315) and the MCA stenosis group (*n* = 97). The demographic characteristics and clinical and biochemical vascular risk factors are shown in Table 1. Because this was a cross‐sectional study, the age of patients in the MCA stenosis group was older than that in the no MCA stenosis group, and the ratio of females were higher in the MCA stenosis group. In accord with the age difference in the two groups, coronary heart disease (*p *=* *0.030) and ischemic stroke (*p *=* *0.040) were more prevalent in the MCA stenosis group. However, there was no significant difference (*p *=* *0.325) in the HCY levels between the two groups.

**Table 1:** Baseline characteristics of patients

Characteristics	No MCA stenosis group (*n* = 315)	MCA stenosis group (97)	*t*/*χ* ^2^	*p*
Age, year (mean ± *SD*)	64.65 ± 12.00	71.61 ± 10.29	−5.157	0.000
Male, *N* (%)	174 (55)	35 (36)	10.888	0.001
Smoking,^a^ *N* (%)	73 (23)	10 (10)	1.006	0.316
Hypertension,^b^ *N* (%)	154 (48)	66 (68)	3.124	0.077
Diabetes mellitus,^c^ *N* (%)	66 (21)	25 (26)	0.626	0.429
History of CHD,^d^ *N* (%)	68 (22)	35 (36)	4.709	0.030
Atrial fibrillation,^e^ *N* (%)	13 (4)	6 (6)	0.645	0.422
History of ischemic stroke,^f^ *N* (%)	82 (26)	40 (41)	4.216	0.040
HbA1c	6.32 ± 1.44	6.57 ± 1.23	−1.538	0.125
Fasting blood glucose	6.01 ± 2.40	6.20 ± 2.32	−0.387	0.699
Uric acid	296.64 ± 77.59	297.44 ± 82.44	−0.088	0.930
TC	4.60 ± 1.10	4.57 ± 1.14	0.297	0.766
TG	1.65 ± 1.11	1.55 ± 1.07	0.807	0.420
LDL‐C	2.33 ± 0.78	2.33 ± 0.79	0.066	0.948
Homocysteine	15.34 ± 8.67	16.30 ± 7.51	−0.985	0.325

*Note*

Abbreviations: CHD, coronary heart disease; LDL‐C, low‐density lipoprotein cholesterol; MCA, middle cerebral artery; TC, total cholesterol; TG, triglyceride.

a Smoking status was recorded as never or not in males.

b Blood pressure ≥140/90 mmHg or self‐report of history of hypertension or current antihypertensive treatment.

c Fasting blood glucose levels >7.0 mmol/L or self‐report of history or antidiabetic drugs or insulin used.

d Self‐report of history of myocardial infarction or angina pectoris and antiplatelet drugs used.

e Diagnosed using electrocardiogram and stethoscope or self‐report of history or anticoagulant drug used.

f Self‐report of history and antiplatelet drugs used.

### 3.2 Association between MCA stenosis and the indicated factors at baseline

To analyze the association between MCA stenosis and the indicated factors, bivariate logistic regression analysis was performed. MCA stenosis was defined as 1, and no MCA stenosis was defined as 0. Males were defined as 1, and females were defined as 0. Smoking, hypertension, diabetes mellitus, coronary heart disease, atrial fibrillation and ischemic stroke were classified as yes or no and defined as 1 or 0, respectively. Similar to the results above, Table 2 showed that there were significant associations between older age (*p *=* *0.001), being male (*p *=* *0.003), hypertension (*p *=* *0.039), ischemic stroke (*p *=* *0.004) and MCA stenosis. However, there was no association between HCY levels and MCA stenosis (*p *=* *0.447).

**Table 2:** Bivariate logistic regression analysis on MCA stenosis for indicated variables at baseline

	Walds	OR	95% CI	*p* Value
Age	11.023	1.045	1.018–1.073	0.001
Males	8.763	0.390	0.209–0.727	0.003
Smoking	0.044	0.914	0.396–2.113	0.834
Hypertension	4.249	1.763	1.028–3.023	0.039
Diabetes mellitus	0.178	0.847	0.392–1.832	0.673
History of CHD	0.484	1.222	0.695–2.147	0.487
Atrial fibrillation	0.066	0.863	0.281–2.656	0.798
History of ischemic stroke	8.269	2.130	1.272–3.656	0.004
HbA1c	0.278	1.078	0.815–1.427	0.598
Fasting blood glucose	0.001	1.002	0.862–1.165	0.976
Uric acid	0.254	1.001	0.997–1.004	0.614
TC	0.030	0.936	0.442–1.984	0.863
TG	0.286	0.592	0.611–1.325	0.592
LDL‐C	0.017	1.070	0.390–2.934	0.895
Homocysteine	0.578	1.012	0.981–1.044	0.447

*Note*

Abbreviations: CHD, coronary heart disease; LDL‐C, low‐density lipoprotein cholesterol; MCA, middle cerebral artery; TC, total cholesterol; TG, triglyceride.

### 3.3 Differences in the indicated factors between the no MCA stenosis group and MCA stenosis group after age and sex matching

Given the influence of age and sex on MCA stenosis and other factors, the two groups were matched for age and sex. As shown in Table 3, the uric acid level in the MCA stenosis group was higher than that in the no MCA stenosis group (*p *=* *0.004) in males. Similar to the results above, there was still no significant difference in HCY levels between the two groups in males (*p *=* *0.540) and females (*p *=* *0.061). For other factors, there was no significant difference between the two groups.

**Table 3:** Characteristics of patients after age and sex macthed

	No MCA stenosis group	MCA stenosis group	*t*/*χ* ^2^	*p*
	Males (*n* = 102)/Females (*n* = 126)	Males (*n* = 35)/Females (*n* = 58)		
Age, year (mean ± *SD*)	72.91 ± 7.52/67.21 ± 9.3	75.69 ± 7.12/70.10 ± 9.65	−1.909/−1.928	0.058/0.055
Smoking, *N* (%)	38 (37)	10 (29)	0.429	0.512
Hypertension, *N* (%)	48 (47)/68 (54)	22 (63)/42 (72)	0.803/1.361	0.370/0.243
Diabetes mellitus, *N* (%)	27 (26)/25 (20)	6 (17)/19 (33)	0.788/2.158	0.375/0.142
History of CHD, *N* (%)	30 (29)/31 (25)	15 (43)/20 (34)	1.031/1.066	0.310/0.302
Atrial fibrillation, *N* (%)	8 (8)/4 (3)	3 (9)/3 (5)	0.016/0.048	0.572/0.682
History of ischemic stroke, *N* (%)	31 (30)/35 (28)	20 (57)/28 (48)	3.355/3.453	0.067/0.063
HbA1c	6.54 ± 1.38/6.37 ± 1.51	6.40 ± 1.09/6.68 ± 1.33	0.560/−1.331	0.576/0.185
Fasting blood glucose	6.12 ± 2.03/6.11 ± 2.38	5.66 ± 1.09/6.56 ± 2.82	1.275/−1.118	0.204/0.265
Uric acid	295.26 ± 72.84/280.63 ± 72.10	337.83 ± 79.46/273.53 ± 77.19	−2.914/0.606	0.004/0.545
TC	4.18 ± 1.00/4.98 ± 1.04	4.25 ± 0.87/4.75 ± 1.28	−0.329/1.224	0.743/0.224
TG	1.32 ± 0.80/1.75 ± 1.04	1.32 ± 0.52/1.63 ± 1.23	−0.010/0.642	0.992/0.522
LDL‐C	2.15 ± 0.68/2.50 ± 0.78	2.21 ± 0.60/2.40 ± 0.89	−0.455/0.819	0.650/0.414
Homocysteine	17.89 ± 10.50/13.11 ± 5.98	19.11 ± 8.92/14.91 ± 6.12	−0.615/−1.887	0.540/0.061

*Note*

Abbreviations: CHD, coronary heart disease; LDL‐C, low‐density lipoprotein cholesterol; MCA, middle cerebral artery; TC, total cholesterol; TG, triglyceride.

### 3.4 Association between MCA stenosis and the indicated factors after age and sex macthing

After the groups were matched for age and sex (Table 4), bivariate logistic regression analysis revealed that uric acid (*p *=* *0.003) and ischemic stroke (*p *=* *0.008) were associated with MCA stenosis in males and ischemic stroke (*p *=* *0.035) was associated with MCA stenosis in females. However, there was still no association between HCY levels and MCA stenosis in males (*p *=* *0.709) and females (*p *=* *0.098). For other factors, there was no significant association with MCA stenosis.

**Table 4:** Bivariate logistic regression analysis on MCA stenosis for indicated variables after age and sex matched

	Males				Females			
	Walds	OR	95% CI	*P*	Walds	OR	95% CI	*p*
Age	1.414	1.042	0.974–1.115	0.234	0.582	1.015	0.976–1.056	0.445
Smoking	0.023	0.919	0.313–2.700	0.878	/	/	/	/
Hypertension	1.605	1.818	0.721–4.586	0.205	4.246	2.250	1.040–4.865	0.039
Diabetes mellitus	0.298	0.679	0.169–2.730	0.585	0.648	1.523	0.547–4.238	0.421
History of CHD	1.309	1.717	0.680–4.334	0.253	0.015	1.051	0.480–2.298	0.902
Atrial fibrillation	0.814	0.456	0.083–2.514	0.367	0.068	1.256	0.227–6.947	0.794
History of ischemic stroke	7.066	3.471	1.387–8.689	0.008	4.445	2.173	1.056–4.470	0.035
HbA1c	0.778	1.251	0.761–2.056	0.378	0.000	0.998	0.692–1.440	0.991
Fasting blood glucose	0.813	0.843	0.582–1.221	0.367	0.001	1.003	0.840–1.198	0.974
Uric acid	8.612	1.010	1.003–1.017	0.003	3.017	0.995	0.990–1.001	0.082
TC	0.263	0.655	0.130–3.305	0.608	0.064	0.886	0.345–2.272	0.801
TG	0.040	1.084	0.492–2.387	0.842	0.257	0.879	0.533–1.449	0.612
LDL–C	0.393	2.062	0.215–19.823	0.531	0.016	1.083	0.310–3.788	0.901
Homocysteine	0.139	0.991	0.944–1.040	0.709	2.736	1.048	0.991–1.108	0.098

*Note*

Abbreviations: CHD, coronary heart disease; LDL‐C, low‐density lipoprotein cholesterol; MCA, middle cerebral artery; TC, total cholesterol; TG, triglyceride.

## 4 DISCUSSION

In this cross‐sectional study, we found that there was no significant difference in HCY levels between the no MCA stenosis group and MCA stenosis group before and after age and sex macthing. After adjusting for other factors, no association between serum HCY levels and MCA stenosis was found. Meanwhile, we also found that ischemic stroke was more prevalent in individuals with MCA stenosis and that uric acid was higher in males with MCA stenosis. After adjusting for other factors, they were all significantly associated with MCA stenosis.

Elevated serum HCY level is considered to be a risk factor for atherosclerotic diseases, such as ischemic stroke and coronary heart disease (Han et al., 2015; Li et al., 2016; Wang et al., 2006). Lowering HCY with B vitamins and folic acid was considered a promising strategy to prevent cardiovascular and cerebrovascular diseases events in patients. However, several clinical trials have revealed that supplements combining B vitamins and folic acid did not have beneficial effects on the incidence of vascular events (Armitage et al., 2010; Bazzano, Reynolds, Holder, & He, 2006; Bonaa et al., 2006; Ebbing et al., 2008; Lonn et al., 2006; Toole et al., 2004). Therefore, the use of B vitamins and folic acid as secondary prevention in patients with vascular diseases was not supported. However, in the primary prevention clinical trials, folic acid supplementation significantly reduced the risk of first stroke (Huo et al., 2015; Qin et al., 2016). Why is lowering HCY useful in primary prevention and useless in secondary prevention? Our study may provide an explanation: HCY is not associated with MCA stenosis, which is a common and typical example of cerebral artery stenosis. In secondary prevention, the proportion of patients with cerebral artery stenosis is higher than that in primary prevention. Because reduction in HCY cannot reverse cerebral artery stenosis to reduce the incidence of first or recurrent stroke, lowering HCY is often useless in secondary prevention and occasionally useless in primary prevention. Researchers believe that initiation of atherosclerosis requires a high HCY concentration, while in the period from atherosclerosis (without stenosis) to stenosis the HCY concentration may be constant. Lowering HCY before initiation of atherosclerosis may have beneficial effects on the prevention of vascular events. In other word, lowering HCY needs to be as early as possible.

There are some differences between our results and those of another study. In the previous study, the author found that hyperhomocysteinemia might be a risk factor for MCA stenosis in asymptomatic residents (Huang et al., 2007). One reason for the differences is the composition of subjects. In the previous study, asymptomatic residents may have a low percentage of atherosclerosis without stenosis, whereas in our study the subjects may have a high percentage of atherosclerosis without stenosis.

Consistent with previous studies, we also found that uric acid was higher in subjects with MCA stenosis. Kumral, Karaman, Orman, & Kabaroglu (2014) demonstrated that higher uric acid level is strongly associated with carotid artery disease. In the article published by Ahn et al., (2018) the authors reported that higher serum uric acid level was associated with an increased risk of intracranial arterial stenosis among middle‐aged females but not males. In our study, the uric acid level in females with MCA stenosis was higher than that in those without MCA stenosis, but no significant difference was observed. Age of subjects, position of arterial stenosis and sample size may be responsible for the differences.

There are several limitations in the study. As this is a cross‐sectional study, there may be bias in the sample choice. We chose patients in the Neurology Department instead of asymptomatic residents for subjects. We believe that when comparing the differences in HCY levels between MCA stenosis or no stenosis, symptomatic patients may be more appropriate than asymptomatic residents. Moreover, the sample size, especially that of patients with MCA stenosis in the study, was small. Future studies including large samples are warranted. Finally, only a Chinese Han population was studied in this report, so the conclusion needs to be verified in other ethnic populations.

In conclusion, this study indicates a lack of association between serum HCY level and MCA stenosis, which may partially explain the negative results of secondary prevention clinical trials focused on lowering serum HCY level with B vitamins and folic acid supplementation to prevent vascular outcomes. Future studies on HCY reduction should focus on primary prevention of ischemic stroke.

## CONFLICTS OF INTEREST

None declared.

## Data Availability

The data that support the findings of this study are available from the corresponding author upon reasonable request.
